# 52-kDa Ro/SSA epitopes preferentially recognized by antibodies from mothers of children with neonatal lupus and congenital heart block

**DOI:** 10.1186/ar1848

**Published:** 2005-11-04

**Authors:** Christine Fritsch, Johan Hoebeke, Hayet Dali, Vincent Ricchiuti, David A Isenberg, Olivier Meyer, Sylviane Muller

**Affiliations:** 1 UPR 9021 Centre National de la Recherche Scientifique, Institut de Biologie Moléculaire et Cellulaire, Strasbourg, France; 2 Division of Endocrinology, Diabetes & Hypertension, Brigham and Women's Hospital, Harvard Medical School, Boston, MA, USA; 3 Centre for Rheumatology, The Middlesex Hospital, University College London, UK; 4 Groupe Hospitalier Bichat-Claude Bernard, Service de Rhumatologie, Paris, France

## Abstract

Neonatal lupus erythematosus is a rare disorder caused by the transplacental passage of maternal autoantibodies. The 52-kDa Ro/SSA antigen (Ro52) ribonucleoprotein represents an antigenic target strongly associated with the autoimmune response in mothers whose children have neonatal lupus and cardiac conduction disturbances, mainly congenital heart block. The objective of this study was to identify putative Ro52/60-kDa Ro/SSA antigen (Ro60) epitopes associated with neonatal lupus and congenital heart block. The reactivity of IgG antibodies present in the sera from mothers with systemic lupus erythematosus and Sjögren's syndrome and in the sera from asymptomatic mothers (a longitudinal study of 192 samples from 66 subjects) was investigated by ELISA using Ro52, Ro60 and 48-kDa La/SSB antigen proteins, as well as 45 synthetic peptides, 13–24 residues long, of Ro52/Ro60 proteins. One to 19 samples collected before, during and after pregnancy were available for each mother. Forty-three disease controls selected randomly and normal sera were tested in parallel. Although no differences were found between Sjögren's syndrome and asymptomatic mothers of group I, who had at least one infant with neonatal lupus, and of group II, who had healthy babies only, significant differences were observed between lupus mothers from both groups. In the former group of lupus mothers, a significantly higher frequency of antibodies to Ro52 peptides 107–122 and 277–292 was observed. Between 18 and 30 weeks of gestation, the period of risk, there was clearly an elevated level of antibodies reacting with Ro52 peptides 1–13, 277–292 and 365–382. Antibodies to Ro52 peptide 365–382 have been shown previously to cross-react with residues 165–185 of the heart 5-HT_4 _serotoninergic receptor, and might be pathologically important. The level of these Ro52 antibody subsets decreased at the end of pregnancy and after delivery. IgG antibodies to Ro52 peptides 1–13, 107–122, 277–292 and 365–382 may therefore represent important biomarkers to predict a complication in pregnant lupus women with Ro52 antibodies.

## Introduction

Neonatal lupus erythematosus (NLE) is a rare, but severe, passively acquired autoimmune syndrome of neonates characterized by cardiac, dermatological, hepatic and hematological manifestations. Autoimmune-associated congenital heart block (CHB) is often detected between 18 and 24 weeks of gestation and, to date, in its complete form, remains irreversible. In contrast, noncardiac manifestations are transient, resolving by 1 year of age without specific treatment [1]. The mothers of these children often have an autoimmune disorder (i.e. systemic lupus erythematosus [SLE] and/or Sjögren's syndrome [SS]), with antibodies against SSA/Ro and/or SSB/La antigens. However, entirely asymptomatic mothers with these antibodies (generally diagnosed after delivery) can also give birth to infants with CHB. Maternal antibodies to the 52-kDa Ro/SSA (Ro52) and 48-kDa La/SSB (La) antigens have been reported to be more strongly associated with CHB than antibodies to the 60-kDa (Ro60) alone [2,3]. The prevalence of CHB in newborns of prospectively followed women with anti-SSA antibodies and known autoimmune rheumatic disease is 2% [4].

Several lines of evidence support the pathogenic role of Ro and La antibodies, which presumably cross the placenta and damage the conduction system of the developing fetus. It is notable, however, that abnormalities are not detectable in maternal cardiac functions despite exposure to the identical antibodies. The pathogenic role of maternal antibodies is poorly understood. Direct implication of Ro/La antibodies has been described in two studies of fatal CHB. Maternal IgG bearing anti-La idiotypes were identified on the surface of fetal cardiac myocytes [5] and Ro antibodies were found in an affected fetal heart [6]. In addition, complete atrioventricular block could be induced in the rabbit and human fetal heart after perfusion of the aorta with anti-Ro52 antibodies [7,8]. These same antibodies inhibited the whole-cell and single-channel L-type Ca^2+ ^channels. The pathogenic role of Ro52 antibodies in the development of CHB was also supported by an experiment using BALB/c mice as a murine model [9].

The association of CHB with maternal autoantibodies to Ro and La antigens might be due to cross-reactions between maternal anti-Ro/La antibodies and fetal cardiac-specific antigens. A possible antigen targeted by La antibodies might be laminin, a major component of the sarcolemmal membrane of cardiomyocytes, which undergoes conformational changes during development (residues EAKLRA are common to La and B1 laminin) [10,11]. Molecular mimicry between these two self-antigens could thus contribute to the pathogenesis of CHB at an early stage during fetal cardiac development. We have more recently identified a cross-reactive B-cell epitope between the Ro52 protein (in residues 365–382) and the heart 5-HT_4 _serotoninergic receptor (residues 165–185), and have demonstrated that these cross-reactive antibodies antagonized the serotonin-induced L-type Ca^2+ ^channel activation on human atrial cells [12]. Further studies showed that antibodies against the human 5-HT_4 _receptor (5-HT_4_-R) have no intrinsic activity on the receptor itself but block receptor activation on human atrial cells by the cognate hormone serotonin [13]. Most importantly, it was also demonstrated that pups from normal female mice immunized with the 5-HT_4_-R peptide 165–185 showed bradycardia, atrioventricular blocks of type I and type II, longer QT intervals, skin rash and neuromotoric problems [14]. We have thus clearly demonstrated that anti-5-HT_4_-R antibodies are associated with neonatal lupus, that they are pathogenic and that they cross-react with the Ro52 antigen [12-14]. It is notable that while the 5-HT_4_-R is not functional in adult rodents, it is expressed in mouse fetal heart [14]. The expression of 5-HT_4_-R in the human fetal heart has also been confirmed [15].

Although many questions remain to be solved to understand the involvement of 5-HT_4_-R in the pathogenicity of CHB, the quest for biomarkers that predict mothers at risk remains an important challenge. In this study, we have tested the sera from 66 mothers (a longitudinal study of 192 samples) in different clinical subgroups for the presence in their serum of IgG antibodies reacting with Ro52, Ro60 and La proteins, as well as with 39 synthetic peptides covering the whole sequence of the Ro52 protein and with six peptides of the Ro60 protein. The appearance of protein and peptide-reactive antibodies was examined regularly in mothers before, during and after delivery. The aim of this study was twofold; first, to determine whether antibodies to the Ro52 peptide 365–382 are important in predicting a complication in pregnant women with Ro antibodies; and, second, to identify other putative Ro52 epitopes associated with neonatal lupus and CHB.

## Patients and methods

### Patients

Two groups of mothers were defined in this study (Table 1). Group I was composed of 41 consecutive mothers who gave birth to 58 children, of whom at least one infant per mother was affected with NLE. In this group, 10 mothers had a lupus, 15 mothers had SS and 16 mothers were asymptomatic. Among the 58 children of these 41 mothers, 45 children had a CHB (14 males, 18 females, 13 unknowns), five children had a cutaneous NLE (one male, four females), and eight children were healthy (three males, four females, one unknown). The diagnosis of complete CHB was based on fetal echocardiography when the diagnosis of CHB was obtained during fetal life, and was based on postnatal electrocardiogram when bradycardia was detected. Among the 41 mothers who gave birth to a child with NLE, four had a pregnancy that resulted in at least one abortion after an *in utero *fetal demise or early death of a baby younger than 6 months. These 41 mothers were aged 19–56 years (average 32.9 years) when their blood was collected, for some of them a long period of time after pregnancy (maximum 12 years). Group II corresponded to 25 mothers with 30 healthy children. In this group, 18 mothers had a lupus and seven mothers had an SS, the mothers were aged 23–42 years (average 33.6 years) and three abortions occurred. Patients selected in our study mainly presented rheumatological and dermatological manifestations.

For patients with SLE, the symptoms were usually mild to moderately severe, and generally no treatment was necessary by the time surrounding pregnancy. Those who needed to be treated were well controlled by either corticosteroids (about 5–20 mg/day Cortancyl^®^) or hydroxychloroquine (Plaquenil^®^). The latter was in most cases interrupted during pregnancy. Only a few mothers (3/22 documented lupus mothers) required a more aggressive treatment such as cyclophosphamide (Endoxan^®^), which was replaced by corticosteroids during pregnancy. Plasmapheresis was realized to prevent CHB when the risk was high. Aspirin was introduced during pregnancy to prevent the risk of thrombosis.

From each mother of groups I and II, 1–19 serum samples were available to study. A total of 192 sera from mothers were tested all together. All of the women gave informed consent to participate, and the study was approved by the Ethics Committee at the Paris Bichat-Claude Bernard Hospital. Disease controls were studied in addition, including the serum from patients with SLE (*n *= 14), with SS (*n *= 15) and with rheumatoid arthritis (RA; *n *= 14) selected randomly (women without indication of pregnancy), and the sera from 24 healthy blood donors were also tested. Lupus patients' sera were randomly taken from the serum collection from one of the authors (DAI) and no pre-selection according to disease manifestation or severity was made. The global score (British Isles Lupus Assessment Group system) used to measure the lupus disease severity of 14 female patients (aged 17–58 years; average 28.4 years) was between 1 and 22 (inactive/mildly active disease, score 1–5, *n *= 6; moderately to severely active disease, score 6 or greater, *n *= 8). Eight of these patients were Caucasian, four patients were Afro-Caucasian and two patients were Chinese. Informed consent was obtained from all participants in accordance with the medical ethical regulations of the local committee. Sera were stored at -70°C until use.

### Ro proteins and Ro synthetic peptides

Affinity-purified Ro60 and La proteins were obtained from Immunovision (ref. SSA-300; Springdale, AR, USA). The production and purification of human recombinant Ro52 (rRo52), a kind gift from G Pruijn and W van Venrooij (Nijmegen, The Netherlands), has been described previously [16]. Synthesis and purification of 39 overlapping peptides of Ro52 and of six peptides of Ro60 have been described previously [16,17]. Ro52 peptides encompassed 13–24 amino acid residues and, in general, overlapped each other by 1–10 residues. Selection of peptides that have been synthesized have also been made according to difficulties of synthesis and/or solubility in aqueous solvents. The Ro60 peptide 21–41 was conjugated to ovalbumin through the -SH group of cysteine 38 using *m*-maleimido benzoyl-*N*-hydroxy succinimide ester as a coupling agent [17].

### ELISA, immunodiffusion and Western blotting

The methods used for testing antibodies reacting with rRo52, Ro60 and La proteins in the three types of assay and Ro52/Ro60 peptides in ELISA have been previously described [16,17]. Only the IgG response was tested in the ELISA and Western blotting assay. Peptides were found to be satisfactorily attached to the plastic surface of ELISA plates using antibodies induced in rabbits against these peptides [18]. For calculations, all optical density (OD) values >3 measured in the ELISA were considered as 3.0. The cutoff points of each assay were determined from a series of sera collected from 24 normal donors using the mean OD values of these normal sera tested in parallel + 2 standard deviations. For convenience, the 192 sera screened with the 48 antigens (proteins and peptides) were considered positive when OD ≥0.3. When this threshold value was used, none of the normal sera was found positive. Mean OD values correspond to the arithmetic mean of all OD values, including values under the cutoff line for positivity. Statistically significant frequency differences between groups were determined by Student's *t *test. Mean OD values differences between groups were analyzed using the Mann-Whitney *U* test. *P *< 0.05 was considered significant.

## Results

### Reactivity of mothers' sera with Ro52/Ro60 proteins and peptides

The sera from mothers with SLE and SS as well as sera from asymptomatic mothers were tested with Ro proteins by immunodiffusion, Western blotting and ELISA. Previous studies have shown that distinct antibody subsets are identified using these different immunoassays [16]. This observation was confirmed in the present study (Table 2). In general, the ELISA detected Ro52 and Ro60 antibodies in a higher number of sera than Western blotting. A number of sera were positive in immunodiffusion tests but negative in ELISA and/or Western blotting tests. Conversely, some immunodiffusion-negative sera were positive in the ELISA and/or Western blotting tests. It is noticeable, however, that Ro antibodies were more frequently detected in the serum from patients with SLE and SS than in the serum from asymptomatic mothers (Table 2). Interestingly most of the sera from SLE, SS and asymptomatic mothers contained antibodies reacting with both Ro52 and Ro60. Very few sera contained antibodies reacting with only one Ro protein (or with La only; data not shown), and this reactivity profile was consistently observed in mothers of both groups I and II. No statistically significant difference was observed between the sera of groups I and II when the reactivity of sera with the Ro52 and Ro60 proteins was examined.

The 192 sera from 41 mothers of group I and from 25 mothers of group II were then tested for their ability to react in the ELISA with 39 overlapping peptides covering the whole Ro52 sequence and with six Ro60 peptides. The reactivity of autoimmune sera with these 45 peptides has been described previously [16,17,19]. The data (frequency and *P *values) presented in Table 3 take into account the number of samples collected from each mother that differs from 1 to 19. The analysis of data showed that, among the 45 peptides tested in ELISA, only three (sequences 191–208, 262–279 and 326–340 of Ro52) were never recognized by IgG antibodies contained in the sera of groups I and II. Thirty-one other peptides were recognized by less than 25% of sera, and 11 peptides were recognized by at least 25% of sera (Table 3).

When the reactivity of SLE sera was analyzed and compared in both groups I and II, we observed first that the spectrum of reactivity of antibodies from mothers of group I was larger (11 peptides versus 5 peptides recognized by at least 25% of sera). Second, we found that the level of IgG antibodies to Ro52 peptide 107–122 (69% versus 28%; *P *= 0.023) and peptide 277–292 (83% versus 49%; *P *= 0.049) and to Ro60 peptide 21–41 (64% versus 27%, *P *= 0.044) was more frequently elevated in the serum from mothers of group I. These differences were statistically significant with *P *≤ 0.050. Some peptides were recognized much more frequently by the antibodies from mothers of group I, although in a non-statistically significant manner; for example, with the Ro52 peptide 107–126 (37% versus 7%; *P *= 0.062) and the Ro52 peptide 365–382 (77% versus 47%; *P *= 0.054).

It is noticeable that in contrast to what was observed with the sera from lupus mothers, the sera from mothers with SS reacted with fewer Ro peptides; namely, four Ro52 peptides (sequences 1–13, 107–122, 277–292 and 365–382) and two Ro60 peptides (sequences 21–41 and 304–324), which were recognized by at least 25% of sera. The percentage of sera positive with these peptides in groups I and II was not statistically significantly different (Table 3).

The sera from asymptomatic mothers with affected children collected 3 months-10 years after delivery (mean 4.8 years) reacted weakly and infrequently with Ro52 and Ro60 peptides (Table 3). The reactivity of sera with La protein follows the same pattern of reactivity; namely, a highly significant elevation of positive sera from lupus mothers of group I as compared with lupus mothers of group II, positivity of sera from mothers with SS that was not statistically different in groups I and II, and a low frequency of positive sera in the group of asymptomatic mothers of group I (Table 3).

If we considered a mother positive when at least one of samples was positive with a given peptide, instead of taking into account the number of serum samples available for each mother, very similar results were obtained. Between group I (*n *= 10) and group II (*n *= 18) of mothers with lupus, statistically different reactivities were found with Ro52 peptide 107–122 (88% versus 33%, *P *= 0.011), peptide 277–292 (100% versus 44%, *P *= 0.007) and peptide 365–382 (100% versus 61%, *P *= 0.039), and with Ro60 peptide 21–41 (75% versus 33%, *P *= 0.049) and peptide 304–324 (75% versus 33%, *P *= 0.049). Reactivities with recombinant Ro52 protein (100% versus 78%), Ro52 peptide 1–13 (88% versus 56%) and La protein (100% versus 50%) were no longer statistically different between groups I and II of lupus patients. No difference was observed between groups I and II of mothers with SS.

In summary, the most significant differences observed between mothers of groups I and II were in lupus mothers of group I, who possessed a significantly higher frequency of IgG antibodies to Ro52 peptides 107–122 and 277–292 and to Ro60 peptide 21–41. In a series of disease control sera collected from randomly selected patients with SLE, SS and RA, the data showed that, in good agreement with our previous results, the two Ro52 peptides were not recognized or were recognized infrequently [16,19] by the sera from these patients.

### Longitudinal study of sera from lupus mothers of groups I and II

**Figure 1:**
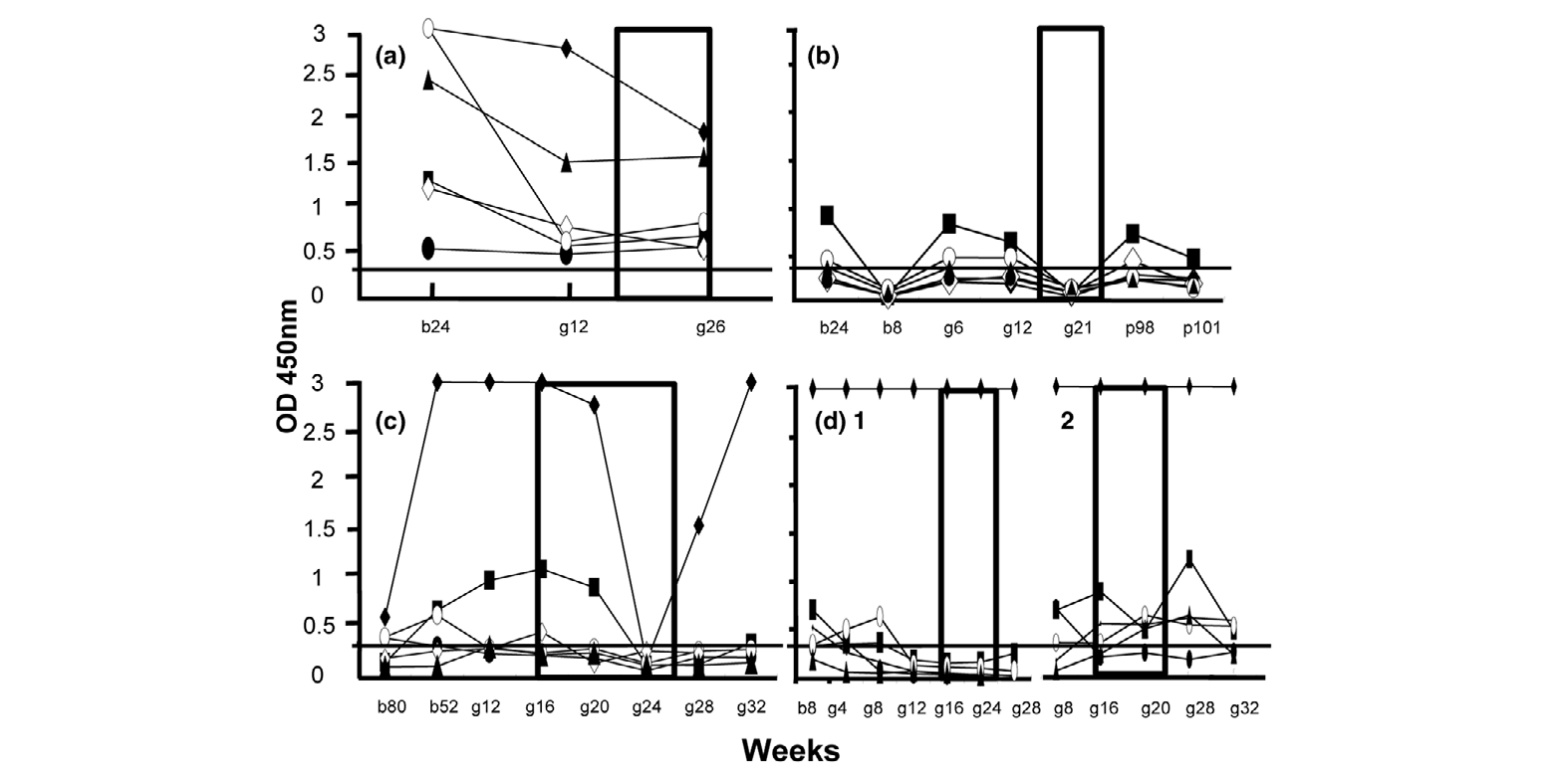
Longitudinal study of sera from lupus mothers of groups I and II with Ro antigens. The sera from lupus mothers of **(a) **group I and **(b)**–**(d) **group II were tested by ELISA before (b), during (g, for gestation) and after (p, for post) pregnancy. The number of weeks is indicated. Mother D had two consecutive pregnancies (D1 and D2). The rectangles represent the period known to be at risk (approximately weeks 16–26 of gestation), knowing that autoimmune-associated congenital heart block most often occurs between 18 and 24 weeks of gestation. Sera were tested at a 1:1,000 dilution. Only the IgG response was evaluated. All samples were systematically tested in at least two independent tests – the results shown in the figure correspond to one complete representative experiment. The sera were considered positive when the optical density (OD) values were ≥0.3 (horizontal lane). All sera were tested with the 48 antigens. Only the Ro antigens showing the highest reactivity are depicted in the figures; ◆, recombinant Ro52; ■, Ro52 peptide 1–13; •, Ro52 peptide 107–122; ◊, Ro52 peptide 277–292; ○, Ro52 peptide 365–382; ▴, La.

**Figure 2:**
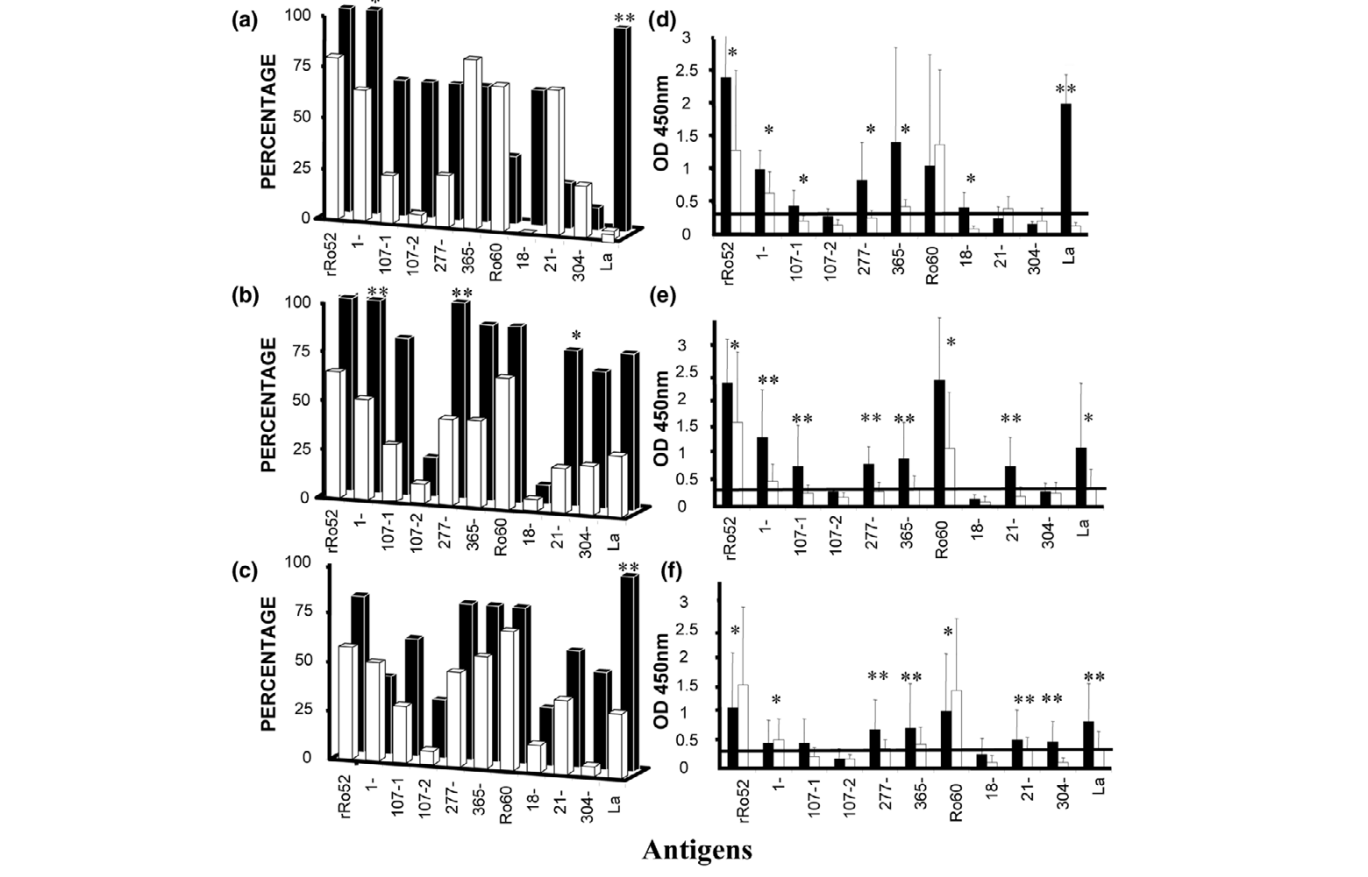
Reactivity in ELISA of sera from lupus mothers of groups I and II with Ro antigens. Sera were classified into three subgroups: **(a)**, **(d) **before, **(b)**, **(e) **during, and **(c)**, **(f) **after pregnancy. The results are expressed in terms of frequency (%) of positive reaction ((a)–(c)) and antibody level (mean optical density [OD] values + standard deviation) ((d)–(f)). Mothers' sera were tested at a 1:1,000 dilution and the IgG response only was evaluated. The number of samples (number of mothers) was as follows; group I (black bars): (a)–(d), 5 (3); (b)–(e), 8 (5); (c)–(f), 6 (5); group II (white bars): (a)–(d), 18 (4); (b)–(e), 44 (16); (c)–(f), 12 (7). All samples were systematically tested in at least two independent tests. The results shown in the figure correspond to one complete representative experiment. The sera were considered positive when OD ≥0.3 (visualized by a longitudinal lane in panels (d)–(f)). All sera were tested with the 48 antigens. Only the Ro antigens showing reactivity are depicted. For clarity, the first residue of each peptide sequence is indicated in the figures (107-1 for 107–122; 107-2 for 107–126). (a)–(c) Student's *t *test, *0.05 > *P *> 0.01, **P ≤ 0.01; (d) and (e) Mann-Whitney *U* test, *0.05 > *P *> 0.005, **P ≤ 0.005.

The data already described indicate that certain antibody subtypes occur more frequently in the serum of lupus mothers of children with NLE than in the samples from lupus mothers with healthy children. The appearance of IgG antibodies to Ro antigens (Ro52, Ro60, La proteins and the 45 peptides of Ro52 and Ro60) was then examined in serial samples from lupus mothers of groups I and II in order to establish, in a more individual manner, whether correlations exist between the presence of these antibody subsets and different parameters related to the disease, the treatment and the course of pregnancy. Figure 1 shows longitudinal testing of sera from four representative mothers. The cumulative data are reported in Figure 2, in which we have subdivided the analysis into three periods: before pregnancy (in a range of 18 months with a mean at 11 weeks for group I, and in a range of 21 months with a mean at 48 weeks for group II; Figure 2a,d), during pregnancy (Figure 2b,e) and after pregnancy (in a range of 26 months with a mean at 21 weeks for the group I, and in a range of 12 months with a mean at 17 weeks for the group II; Figure 2c,f). In a general manner, whichever period is examined and for most of the antibody specificities, the level of IgG antibodies (expressed as mean OD values) and their frequency (%) were clearly higher in the mothers of group I as compared with mothers of group II. This observation is true for antibodies reacting with several Ro peptides and for the antibodies to whole Ro proteins (most particularly for La and Ro52 antibodies). Before pregnancy, the levels of antibodies to Ro52 peptides 277–292 and 365–382 (in terms of mean OD values) as well as Ro52 peptides 1–13, 107–122 and 107–126 (in terms of frequency) were particularly elevated in group I. During pregnancy the same antibodies except the antibodies to Ro52 peptide 107–126 were also elevated, and after pregnancy antibodies to Ro52 peptides 277–292 and 365–382 were elevated.

**Figure 3:**
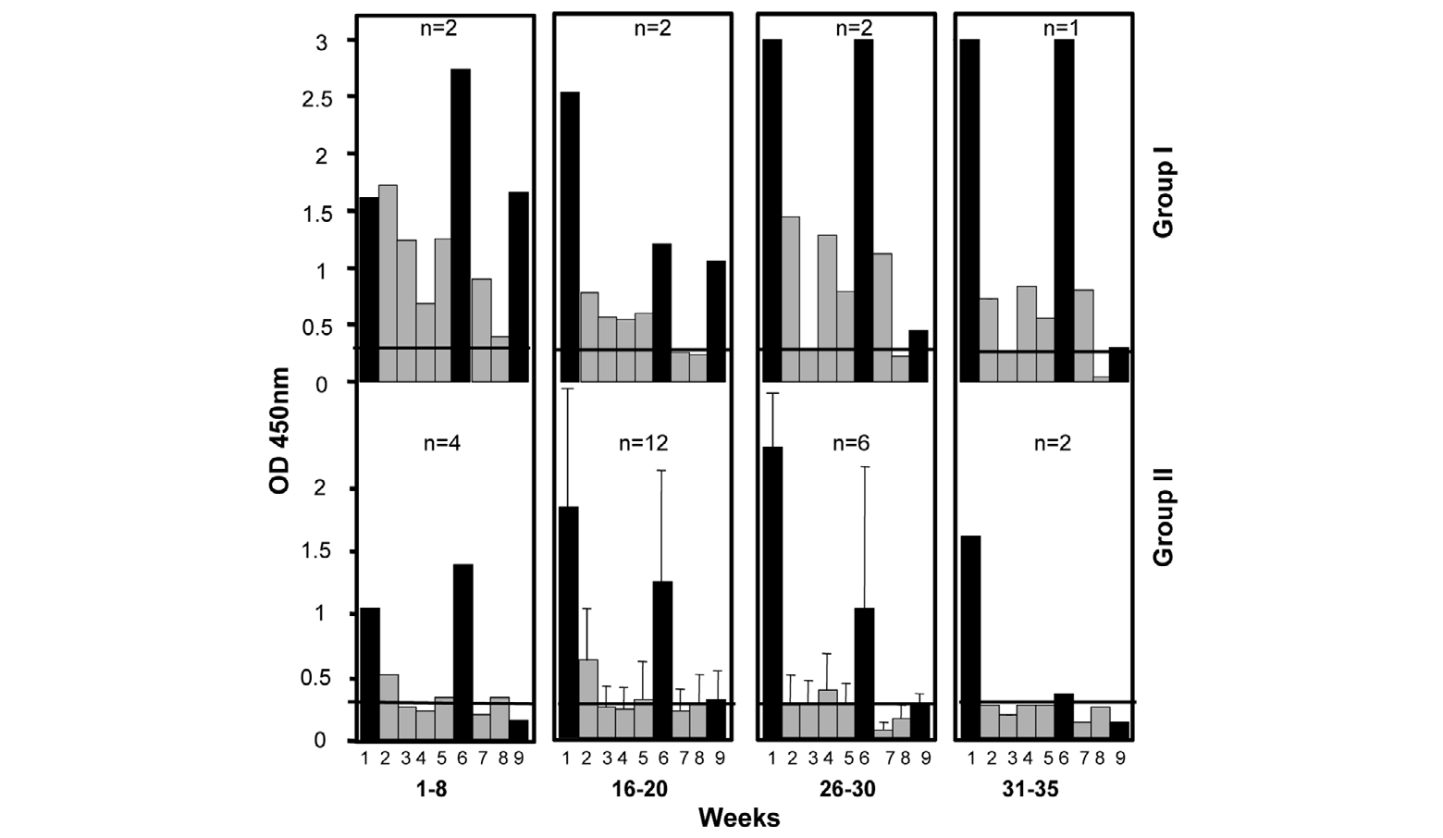
Reactivity in ELISA of sera collected from lupus mothers of groups I and II during pregnancy. Sera were classified in four subgroups, corresponding to weeks 1–8, 16–20, 26–30 and 31–35 of gestation. The number of sera in each subgroup is indicated (*n*). Sera were tested at a 1:1,000 dilution. The IgG response only was evaluated. The sera were considered positive when OD ≥0.3 (horizontal lane). All sera were tested with the 48 antigens. Only the Ro antigens showing reactivity are depicted in the figures. Antigens tested: columns 1, 6 and 9 (black bars), recombinant Ro52, Ro60 and La proteins, respectively; columns 2–5 (grey bars), Ro52 peptides 1–13, 107–122, 277–292 and 365–382; columns 7 and 8, Ro60 peptides 21–41 and 304–324.

In Figure 3, the analysis was further focused on the pregnancy period and the data were examined by defining four testing periods: weeks 1–8, weeks 16–20, weeks 26–30 and weeks 31–35 of pregnancy. Seven samples from five mothers and 24 samples from 17 mothers were examined for group I and group II, respectively. The major features of this study were that the overall level of antibodies was higher in the mothers of group I as compared with group II, and that between 16 and 30 weeks of gestation, the period of risk, there was clearly an elevated level of antibodies reacting with Ro52 peptides 1–13, 277–292 and 365–382.

One of our patients with high levels of IgG antibodies reacting with Ro60, rRo52, Ro52 peptides 1–13, 107–122, 277–292 and 365–382, and Ro60 peptide 21–41 was treated by plasmapheresis. This lupus mother had a first baby with CHB. Plasmapheresis was used for her second pregnancy as a method of treatment aimed at removing antibodies from her plasma to decrease potential autoimmune activity after placental passage. IgG antibodies were efficiently removed from the bloodstream as measured in serum samples collected sequentially during her pregnancy. The mother gave birth to a healthy child.

## Discussion

The antigenic structure of Ro52, Ro60 and La has been extensively studied by several independent groups, and a number of dominant epitopes have been identified in these proteins [20-22]. However, probably because the number of available sera is relatively low, there are very few systematic mapping studies aimed at the characterization of particular regions of Ro and La antigens recognized by CHB-associated antibodies from mothers of affected infants or, in this context, from children suffering from NLE [11,23]. Studies with Ro antigens reported the reactivity of selected sera with recombinant fragments tested by ELISA and Western blotting assay, and it was found that anti-Ro reactivity in mothers of infants with CHB reacted mainly against the Ro52 sequence 200–239, whereas the predominant activity in control mothers was against the sequence 176–196 [23]. This result was confirmed with synthetic peptides, and the most recent study demonstrated that pups born from rats immunized with the Ro52 peptide 200–239 developed atrioventricular block [24]. In the present study, synthetic peptides 13–24 residues long were used in an ELISA to analyze the Ro52 and Ro60 antibody response in mothers whose children have NLE. The whole proteins Ro52, Ro60 and La were also included in this study. The 192 sera from a total of 66 mothers in the two different clinical groups I and II (with or without infants with NLE) as well as the 43 disease controls were studied. The appearance of protein and peptide-reactive antibodies was examined before and during the time of pregnancy, as well as after delivery.

A thorough mapping of autoantibody specificities was undertaken. The high frequency of IgG antibodies to the whole proteins Ro52, Ro60 and La in all selected sera was notable, but a large number of discrepancies between the results obtained via immunodiffusion, Western blotting and ELISA was observed. This lack of correlation has been described previously and may be due, for example, to the absence of Ro52 in total test antigen used in immunodiffusion or to the fact that the Ro antigen used was extracted from bovine spleen, which is known to be less effectively recognized by patients' autoantibodies than human Ro [25,26].

A second set of data was obtained by examining the reactivity of sera with 45 peptides of Ro52 and Ro60 proteins. Eleven Ro peptides were recognized by IgG antibodies from at least 25% of mothers included in this study. These peptides encompass residues 1–13, 50–64, 93–116, 107–122, 107–126, 236–250, 277–292, 365–382 of Ro52 and residues 18–38, 21–41 and 304–324 of Ro60. Although the actual fragments 176–196 and 200–239 of Ro52 described by Wahren-Herlenius and colleagues [23,24] were not tested in this study, thus precluding any direct conclusion, we noted infrequent reactivity in the four peptides covering these regions (Ro52 peptides 178–193, 191–208, 206–224, 222–235). While the reactivity of sera with the whole proteins Ro52, Ro60 and La was high in subgroups of SLE and SS mothers and did not help to distinguish the group of mothers at risk, the overall level of IgG antibodies reacting with several Ro peptides, however, was significantly higher in lupus mothers of group I as compared with group II. These peptides encompass residues 107–122 and 277–292 of Ro52 and residues 21–41 of Ro60. Antibodies reacting with Ro52 peptides 1–13 and 365–382 were also particularly elevated in lupus mothers of group I. In contrast, no statistically significant difference was observed between groups I and II of mothers with SS, and low levels of Ro peptide reactive antibodies were measured in asymptomatic mothers who gave birth to babies with NLE.

The lack of significant association of any antibody subset in groups I and II of patients with SS is intriguing. It is not known whether this result suggests that there are different physiopathological mechanisms involved in SLE and SS. This important question will be examined further in a larger group of mothers. On the other hand, since La reactivity within the group of mothers with SLE seems to be significantly more prevalent in group I compared with group II (Table 3), another envisaged study will be to evaluate the reactivity of mother's sera with overlapping La peptides.

An important feature demonstrated in this study was that, during the critical window of pregnancy between 18 and 30 weeks of gestation, there was a high level of IgG antibodies reacting with Ro52 peptides 1–13, 277–292 and 365–382 in lupus mothers of group I. This result is particularly important to highlight because these antibodies might play an important role in the pathogenesis of CHB at an early stage during fetal cardiac development, and also because the Ro52 sequence 365–382 does correspond to the site of homology with the serotoninergic 5-HT_4_-R [12-14]. It has been confirmed recently that IgG antibodies reacting with peptide 165–185 of 5-HT_4_-R effectively occurred in a higher proportion of mothers whose children had CHB [27]. Although a meta-analysis should be performed to confirm the data, this study raises the point that tracing IgG antibodies reacting with peptide 365–382 of Ro52 might be important in patients with lupus to follow at-risk pregnancies.

Our previous studies have provided some important features concerning the pathological consequences for the fetal heart of antibodies to 5-HT_4_-R [12-14]. It would be of interest to prolong this study by demonstrating the possible physiopathological role of Ro antibodies reacting with Ro52 peptide 365–382, and to determine whether the other antibody subsets of Ro52 antibodies (antibodies reacting with Ro52 peptides 1–13, 107–122 and 277–292, for example) also generate electrophysiological disturbances compatible with CHB.

## Conclusion

Longitudinal analysis of mothers with SLE and SS showed that in the group of lupus mothers whose at least one child had NLE, a significantly higher frequency of IgG antibodies to Ro52 peptides 107–122 and 277–292 was observed. Between 18 and 30 weeks of gestation, the period of risk, there was clearly an elevated level of circulating antibodies reacting with Ro52 peptides 1–13, 277–292 and 365–382. This result is particularly important since antibodies to Ro52 peptide 365–382 have been shown previously to cross-react with residues 165–185 of the heart 5-HT_4 _serotoninergic receptor and might be pathologically important. The level of these Ro52 antibody subsets decreased at the end of pregnancy and after delivery. IgG antibodies to Ro52 peptides 1–13, 107–122, 277–292 and 365–382 could therefore be important to predict a complication in pregnant lupus women with Ro52 antibodies.

## Abbreviations

CHB = congenital heart block; ELISA = enzyme-linked immunosorbent assay; 5-HT_4_-R, 5-HT_4 _receptor; La, 48-kDa La/SSB antigen; NLE, neonatal lupus erythematosus; OD, optical density; Ro52, 52-kDa Ro/SSA antigen; Ro60, 60-kDa Ro/SSA antigen; SLE, systemic lupus erythematosus; SS, Sjögren's syndrome.

## Competing interests

The authors declare that they have no competing interests.

## Authors' contributions

CF performed and analyzed the immunoassays. JH was involved in the analysis of the immunoassay results. HD performed and analyzed the immunoassays. VR performed and analyzed the immunoassays. DAI collected the patient sera and provided patient data. OM conceived of the study, participated in its design, collected the patient sera and provided patient data. SM conceived of the study and was involved in its design and coordination. All authors read and approved the final manuscript.

## Figures and Tables

**Table 1:** Description of the study groups

Symptom	Group I		Group II		Total	
	
	Mothers	Children	Mothers	Children	Mothers	Children
Systemic lupus erythematosus	10 (24)	13	18 (91)	21	28 (115)	34
Sjögren's syndrome	15 (17)	26	7 (40)	9	22 (57)	35
Asymptomatic	16 (20)	19	0	0	16 (20)	19
Total	41 (61)	58	25 (131)	30	66 (192)	88

Numbers in parentheses indicate the number of serum samples tested in each group.

**Table 2:** Reactivity in different tests of sera from mothers of groups I and II

Mothers	Immunodiffusion	Western blotting		ELISA	
		
	Ro (%)	Ro52 (%)	Ro60 (%)	Ro52 (%)	Ro60 (%)
Systemic lupus erythematosus					
Group I (*n *= 9 mothers; 12 samples)	25	67	25	92	67
Group II (*n *= 14 mothers; 26 samples)	69	12	23	62	69
Total (*n *= 23 mothers; 38 samples)	47	39	24	77	68
Sjögren's syndrome					
Group I (*n *= 14 mothers; 14 samples)	71	57	29	64	64
Group II (*n *= 6 mothers; 12 samples)	100	50	25	100	100
Total (*n *= 20 mothers; 26 samples)	86	54	27	82	82
Asymptomatic					
Group I (*n *= 13 mothers; 13 samples)	38	23	8	31	38

The results are shown for mothers, the serum of whom has been systematically tested in the three assays. Only the IgG antibody response was tested in Western blotting and ELISA. The first figure in parentheses represents the number of mothers and the second represents the total number of samples tested. The data take into account the number of samples collected from each mother, which differs from 1 to 5. Thus, for each mother, the frequency of positivity (%) to each peptide was determined as the number of positive sera reported to the total number of samples.

**Table 3:** Reactivity in ELISA of sera from mothers of groups I and II

Antigen	Systemic lupus erythematosus			Sjögren's syndrome			Asymptomatic
	
	Group I (%)	Group II (%)	*P*	Group I (%)	Group II (%)	*P*	Group I (%)
Recombinant Ro52 protein	97	66	0.009*	67	98	0.027*	31
1–13 MASAARLTMMWEE	77	46	0.084	47	58		31
10–25 MWEEVTPICLDPFVE	0	14		0	0		0
12–35 EEVTCPICLDPFVEPVSIECGHSF	10	16		20	8		13
25–40 EPVSIECGHSFCQECI	0	13		10	0		6
34–56 SFCQECISQVGKGGGSVCAVCRQ	0	1		7	0		13
38–53 ECISQVGKGGGSVCAV	3	4		7	0		0
50–64 VCAVCRQRFLLKNLR	25	3		13	5		6
63–79 LRPNRQLANMVNNLKEI	0	6		0	3		0
77–94 KEISQEAREGTQGERCAV	18	1		3	0		0
93–116 AVHGERLHLFCEKDGKALCWVCAQ	40	12		10	9		0
94–109 VHGERLHLFCEKDGKA	3	13		0	0		0
107–122 GKALCWVCAQSRKHRD	69	28	0.023*	47	33		13
107–126 GKALCWVCAQSRKHRDHAMV	37	7	0.062	13	8		13
121–137 RDHAMVPLEEAAQEYQE	0	4		0	0		0
135–151 YQEKLQVALGELRRKQE	3	14		0	0		0
149–166 KQELAEKLEVEIAIKRAD	0	12		0	1		0
164–180 RADWKKTVETQKSRIHA	0	9		0	0		6
178–193 IHAEFVQQKNFLVEEE	3	14		0	0		0
191–208 EEEQRQLQELEKDEREQL	0	0		0	0		0
206–224 EQLRILGEKEAKLAQQSQA	0	1		7	0		9
222–235 SQALQELISELDRRCHS	17	3		23	1		6
236–250 CHSSALELLQEVIIV	27	6		10	7		0
249–264 IVLERSESWNLKDLDI	0	5		7	0		0
262–279 LDITSPELRSVCHVPGLK	0	0		0	0		0
277–292 GLKKMLRTCAVHITLD	83	49	0.049*	40	24		6
290–304 TLDPDTANPWLILSE	0	7		7	0		0
302–317 LSEDRRQVRLGDTQQS	10	2		7	0		0
314–328 TQQSIPGNEERFDSY	0	9		0	9		0
326–340 DSYPMVLGAQHFHSG	0	0		0	0		0
337–352 FHSGKHYWEVDVTGKE	23	11		3	12		6
350–367 GKEAWDLGVCRDSVRRKG	0	1		0	0		9
365–382 RKGHFLLSSKSGFWTIWL	77	47	0.054	50	33		25
380–396 IWLWNKQKYEAGTYPQT	0	5		0	2		0
394–412 PQTPLHLQVPPCQVGIFLD	18	1		3	10		0
411–423 LDYEAGMVSFYNI	0	2		0	0		0
422–438 NITDHGSLIYSFSECAF	16	2		0	0		0
436–451 CAFTGPLRPFFSPGFN	13	7		13	10		0
447–463 SPGFNDGGKNTAPLTLC	8	9		0	6		0
463–475 CPLNIGSQGSTDY	0	7		7	1		0
Ro60 PROTEIN	80	67		67	99	0.022*	31
1–23 MEESVNQMQPLNEKQIANSQDGY	15	6		17	1		6
18–38 NSQDGYVWQVTDMNRLHRFLC	32	11		20	1	0.097	0
21–41 DGYVWQVTDMNRLHRFLCFGS	64	27	0.044*	23	38		0
304–324 VCEKLCNEKLLKKARIHPFHI	50	18		27	4		13
495–518 KLIVCGMTSNGFTIADPDDRGMLD	10	24	0.058	7	11	0.079	13
524–538 TGALDVIRNFTLDMI	10	17		3	9		0
La PROTEIN	93	30	<0.0001*	40	68		13

The 192 samples described in Table 1 were systematically tested in at least two independent tests. Only the IgG response was tested. Patients' sera were diluted 1:1,000. To be tested in the ELISA, peptide 21–41 of Ro60 protein was conjugated to ovalbumin. The sera were considered positive when optical density ≥0.3. Statistically significant differences between groups were determined by Student's *t *test. *P < 0.05 considered significant.
